# Parish apprenticeship and the old poor law in London^1^

**DOI:** 10.1111/j.1468-0289.2009.00485.x

**Published:** 2010-11

**Authors:** Alysa Levene

**Affiliations:** Oxford Brookes University

## Abstract

This article offers an examination of the patterns and motivations behind parish apprenticeship in late eighteenth- and early nineteenth-century London. It stresses continuity in outlook from parish officials binding children, which involved placements in both the traditional and industrializing sectors of the economy. Evidence on the ages, employment types, and locations of 3,285 pauper apprentices bound from different parts of London between 1767 and 1833 indicates a variety of local patterns. The analysis reveals a pattern of youthful age at binding, a range of employment experiences, and parish-specific links to particular trades and manufactures.

Parish apprenticeship has recently been the subject of newly systematic investigation. Prior to the work of scholars like Honeyman, Humphries, and Hindle, the subject was either discussed in an impressionistic way, or tended to focus on the growth of factory labour.^2^ With the exception of Kirby's study of child labour in Britain, work that highlighted the mixed economy of juvenile labour has consisted of local or small-scale studies which have not overtly tied the state of children's work to wider questions concerning the labour market or economic growth.^3^ Hindle's important work on the sixteenth and seventeenth centuries has now set up a much more rigorous framework for thinking about the origins of parish apprenticeship, which must inform any future investigations.^4^ Honeyman and Humphries et al. have tied patterns of child labour to the process of industrialization, the survival of poor families, and the creation of human capital.^5^ This has put the place of child labour in an industrial workforce on a proper footing for wider reflection. This article represents something of a bridge between these two thrusts of current work. It offers a systematic study of parish apprenticeship by London parishes, and examines how the institution was used by parish officials. It tests the characteristics of parish apprentices in terms of age, type of labour, and location of binding, and relates this to the priorities and aims of the old poor law and the pragmatic concerns of its officers. It picks up the long-standing aims of apprenticeship as set out in and developed from Elizabethan vagrancy and poor laws, and suggests that these aims were still central right through to the end of the old poor law. The development of an industrial workforce, however, simultaneously offered opportunities for parish officials to dispose of large numbers of children at one time. This article thus argues that by taking up these opportunities, parish officials were indeed facilitating the growth of a flexible and specialized factory labour force beyond the capital, as Honeyman and Humphries have suggested.^6^ However, in terms of *aims* and *forms*, they were applying old practices to changed labour needs. This represents a significant widening out of the recent work on pauper apprenticeship, in particular by relating participation in both traditional and industrializing sectors of the economy to a common framework of pauper child labour. While Honeyman's recent work has made valuable links between apprenticeship and factory work, and between child labour and the industrial workforce more generally, this article approaches the topic from the standpoint of the aims and concerns of the old poor law at a time of rapid economic change. Just as the origins of industrialization are now thought to be slower moving and more gradual than the early proponents of ‘revolution’ asserted, so, it will be argued, were the changes in ideas on child labour and its form from the *supply* end.^7^ Apprenticeship towards the end of the old poor law period thus served variously both its long-standing function in providing traditional craft-type training, and the emergence of paid child labour. In many cases, indeed, the distinction was probably not so clear-cut in the minds of parish officers.

The dual nature of the English economy at this time has long been highlighted, of course.^8^ Despite the rapid growth of certain industrializing sectors (most notably cotton), the ‘traditional’ sector continued to make up the lion's share of Britain's economy throughout this period and beyond.^9^ Kirby has highlighted how far this was reflected also in the composition of children's work, and it is notable too in local studies which take in parish apprenticeship.^10^ Several authors have even noted how much child labour in factories and mills owed to the earlier-established tradition of craft apprenticeship.^11^ This article confirms Kirby's findings, but it goes further in demonstrating variability within London itself. London is frequently taken as a case apart from the rest of the country, and it is certainly true that it was not a leader in industrial growth in the new sectors. Despite its impact as a market, a port, and a net consumer of humans, London's economy continued to be dominated by small-unit manufacture and crafts.^12^ As this study will show, however, most pauper children were bound apprentices outside the capital, yet this did not necessarily mean that they moved into sectors which would have been unfamiliar to Londoners. City, Middlesex, and Westminster parishes will be shown to have had notable differences in the types and locations of employment to which they sent their children, which are interpreted in terms of local information networks, employment types, and (to a more limited extent) ideas on child labour. London was able to support a range of traditional sectors while also facilitating the development of regional economies elsewhere. This parish-based variability in the nature of engagement with child labour in different economic sectors has not hitherto been illustrated so systematically.

This finding in turn permits a finer reading of the reasons behind the patterns of parish apprenticeship from London. It is suggested that there was a large degree of pragmatism at work, with parish officials taking up employment opportunities where they could. Apprenticeship was primarily a way to ensure a child's future as a working adult, but this was related severally to paternalist concerns, worries about the future rate burden for the parish and the perpetuation of idleness, and investment in human capital in a wider sense. Here, too, it is likely that parish officials were still thinking in terms of settlement law and employment prospects, and applying a longstanding model of child labour and training to a changing and mixed economy. Even in their dealings with factory masters, parish officials continued to ask the same questions about skills and long-term employability as they had previously, implying a preoccupation with sustainable employment prospects and human capital investment.^13^ As Hindle is at pains to stress, parish apprenticeship formed part of a commitment to creating labour discipline among the poor, and was the only part of the approach to work-creation with any lasting success. Indeed, he has argued that apprenticeship was preferred above the giving of parish relief to adult paupers as a measure to alleviate poverty.^14^ Continuity was again the theme, albeit feeding in to a new area of labour in some cases, and apprenticeship was still a preferable and more rational form of training for parish officials than paid labour.^15^ Factory masters were probably much more aware of the benefits of pauper labour for the changed needs of an industrializing economy than parish officials. These benefits included the intensity of hours that could be demanded, the flexibility of labour, the lack of parents nearby, and (as Humphries has highlighted in particular), the reduced transaction costs it brought in re-educating the workforce in new patterns of discipline and skills.^16^ For parish officials, apprenticeship to factory and traditional sectors alike played into a broader range of co-existing concerns about children's futures and the state of parish finances.

This relationship between current training and future burdens on the poor law is characteristic of the consideration of pauper children as distinct from waged child labourers or the non-poor.^17^ In a broader sense, however, poor children form a significant topic for study for several other reasons. Firstly, the life-course of pauper children had been intimately tied to apprenticeship training since the sixteenth century.^18^ Setting poor children to work was a way to lift not only the child but also his or her family out of indigence, and set the child on a path to future independence. As suggested above, employment and training were not only a pragmatic response to local poverty, but also a way of investing in the human capital of the parish for the future. While officials believed that they were acting in the children's best interests, however, it was not necessarily in accordance with their parents' wishes, making the issue of human capital formation harder to assess.^19^ Nonetheless, Humphries sees this intervention in family life as being ultimately to the benefit of the child and to society.^20^ After apprenticeship became a ‘head of settlement’ in the 1692 Settlement Act, it also became a way to pass on responsibility for the potentially poor to another parish.^21^ At this stage, then, human capital formation may have become a lesser priority than the ordering of the potential rate burden. While the legal basis for parish apprenticeship was the same as for privately bound children, it was tied to a much wider set of concerns about vagrancy, belonging, and future rates of work and dependency.

The second distinctive feature of pauper apprenticeship compared with its privately negotiated counterpart is that it was directed by officials much more than by parents. Pauper parents had little room for negotiation in the last instance, and could theoretically be deprived of relief if they refused to allow their child to be bound.^22^ This means that parish apprenticeship was closely tied to the state of the labour market, at least at its lower end. Parish officials were likely to bind children where they could, and so an investigation of employment patterns is revealing of where opportunities lay, and which trades were inclined to deal with paupers rather than private apprentices. Parents looking for placements for their children might have a much greater focus on aspirations for social advancement, and could choose to enter the market at whatever level they could afford (since premiums were on a sliding scale). An investigation of parish apprentices thus illuminates a different set of questions about the labour market, and arguably spotlights its most pressing areas of expansion (with a need for flexible workers) and contraction (where opportunities were less attractive for parents). Finally, parish apprenticeship is worthy of separate study because it was numerically so significant, especially as a greater proportion of children began to survive into adulthood from the mid-eighteenth century onwards.^23^ For all these reasons, parish apprentices deserve to be studied as a separate group of child workers.

Parish apprenticeship was thus tied to a long-held set of notions about children's work, the encouragement of self-sufficiency among paupers (which relates to the value of investing in human capital at a young age), and settlement. This long pedigree forms an important backdrop to the argument for continuity developed here. Parish officers used the same indentures and negotiated for the same terms for factory apprentices as they had for craft and traditional manufacturing placements for decades. While apprenticeship as a formal system of training is judged by several historians to have been in decline by the late eighteenth century, nevertheless it arguably remained as important as ever at the lower end of the socio-economic scale because of its ties to ideals on poverty prevention, settlement acquisition, and training in skills.^24^ The following sections will present an analysis of 3,285 children apprenticed by London parishes between 1767 and 1833. It will outline their characteristics and tie these to wider considerations of local and more far-flung labour markets, and the aims and concerns of poor law officers.

## I

As stated above, the analysis of parish apprentices will focus on their age, the type of work sector they were bound to, and its geographical location. This will reveal the extent of the role of children's work in the industrializing economy, and whether it was focused in any particular sector. The analysis of age patterns at the time of binding will be suggestive of attitudes towards child labour from both potential masters and binding authorities. The binding of young children suggests that very juvenile labour was profitable for masters, or at least that any disadvantages were outweighed by the long period of their service. It will also suggest how far parish officials prioritized the passing on of responsibility for children as early as possible, and thus hint at the importance of human capital investment over financial concerns. The types of trades commonly used for pauper bindings will direct the spotlight to continuity or change in the occupational profiles of child labour, which has an impact on our perceptions of the pace of industrial growth. Lastly, the regional patterns of parish apprenticeships from London tell us about the impact of the capital on the local pace of industrialization, and the extent to which different parishes maintained links with different economic sectors.

Any systematic examination of the characteristics of London's parish apprentices requires a large quantitative dataset. This is so that trends remain statistically meaningful when broken down by parish and by employment type. Although apprenticeship indentures are the most common source of information on training, a more uniform set of information is available for this period in apprenticeship registers. Comparison was made with surviving indentures to see if extra information on training type was available, but the latter were not found to be more informative for parish children, and were more time-consuming to collate. Registers instead have a largely uniform format across parishes, especially after 1802, when a parliamentary directive mandated London parish officials to keep better records of parish apprentices.^25^ Many had in fact done so in the aftermath of ‘Hanway's Act’ of 1767, which initiated better record-keeping for poor infants.^26^ The current sample was designed to offer as even coverage as possible of parishes in Middlesex, Westminster, and the City, and was based on registers that included 100 or more children. It cannot claim to be entirely exhaustive, but it covers a wide range of parishes across most of London. It is possible that other parishes had their own patterns of apprenticeship, but the current sample is large and geographically wide enough to allow robust conclusions to be drawn. The only area that is regrettably under-represented is the intramural area of the City of London, where parishes bound out too few children to allow for meaningful analysis.^27^ The data recorded in the sample for all children consist of name, age, and master's name, trade, and place of residence.

**Figure 1:**
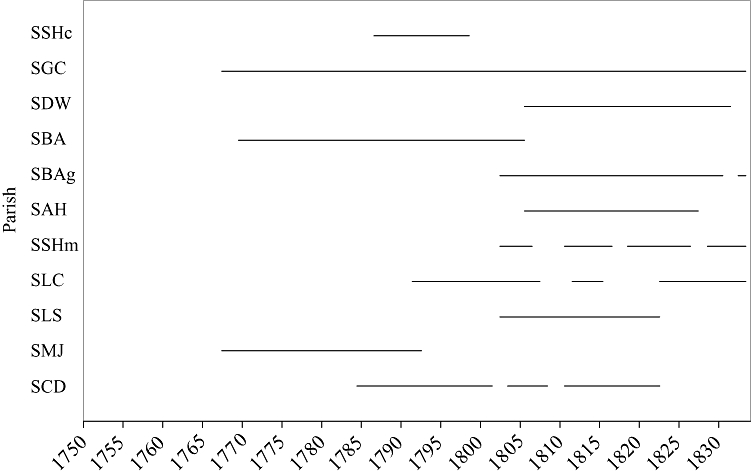
Parish and charity apprenticeship database, 1751–1833: periods of coverage *Notes:* SSHc: St Sepulchre Holborn (City); SGC: St Giles Cripplegate; SDW: St Dunstan in the West; SBA: St Botolph Aldgate; SBAg: St Botolph Aldersgate; SAH: St Andrew Holborn; SSHm: St Sepulchre Holborn (Middlesex); SLC: St Luke Chelsea; SLS: St Leonard Shoreditch; SMJ: St Margaret and St John; SCD: St Clement Danes. *Source:* Apprentice database.

The breakdown of the overall sample of 3,285 children is given in table 1, and the periods of coverage for each parish are shown in figure 1. They indicate the range of geographical coverage achieved across London. St Clement Danes and St Margaret and St John in Westminster, St Leonard Shoreditch in the poorer East End, St Luke Chelsea to the west, and St Sepulchre Holborn (Middlesex division) all come from the Middlesex part of the city. The Westminster area, with its large population of lawyers and courtmen, was known for its wealth in this period, although richer areas tended also to house pockets of the poorer sorts who provided services to the wealthy.^28^ The East End, in contrast, was generally more impoverished, and dependent principally on the seasonal cloth-making trade.^29^ St Andrew Holborn, St Botolph Aldersgate, St Botolph Aldgate, St Dunstan in the West, St Giles Cripplegate, and St Sepulchre Holborn (City division) all lie within the City, although all were extramural and bordered the outside of the city walls. Several fall within the area characterized by Landers as being generally unhealthy and prone to crisis mortality in the second half of the eighteenth century.^30^ The investigation of apprenticeship characteristics by originating parish will show whether the generally higher levels of poverty in these parishes resulted in a different pattern of apprenticing than the richer Westminster area, or the poor East End parish of St Leonard Shoreditch.

**Table 1:** Parishes included in apprentice database, 1751–1833

Parish	N
Middlesex	
St Clement Danes	630
St Margaret and St John Westminster	488
St Leonard Shoreditch	324
St Luke Chelsea	311
St Sepulchre Holborn (Middlesex division)	94
City	
St Andrew Holborn	150
St Botolph Aldersgate	144
St Botolph Aldgate	300
St Dunstan in the West	112
St Giles Cripplegate	622
St Sepulchre Holborn (City division)	110
Total	3,285

*Sources:* Parish registers of apprentices: London Metropolitan Archive: St Leonard Shoreditch (vol. 1), P91/LEN/1332, St Luke Chelsea, P74/LUK/116; Westminster Archive Centre: St Clement Danes, B1266, B1267, and B1268, St Margaret Westminster and St John Westminster, E2566; Guildhall Library: St Andrew Holborn, MS 9602, St Botolph Aldersgate (vol. 1), MS 1471, St Botolph Aldgate, MS 2658, St Giles Cripplegate (vols. 1–2), MS 6096, St Sepulchre Holborn, Middlesex division, MS 9107, St Sepulchre Holborn, City division, MS 3139/9, St Dunstan in the West, MS 3003.

As figure 1 illustrates, the period of coverage differs somewhat between individual parishes. This relates directly to the survival of registers, and is at its fullest in the early nineteenth century. This probably relates to the directive for all parishes to keep registers from 1802. St Giles Cripplegate, St Botolph Aldgate, and St Margaret and St John Westminster had all started keeping registers by the 1760s, although the latter two parishes did not continue them throughout the period studied here. It should thus be noted that numbers of parish children apprenticed per year is at least partly a function of the composition of the dataset rather than a true reflection of changing numbers of children bound in London. Data were only recorded up to the end of 1833 (the last year of the old poor law) even if the registers continued beyond that date.

Of the 3,285 children in the dataset, 42.2 per cent (1,386 cases) were girls. It is worth stressing this feature early on, since it affords a rare opportunity to examine the employment of young females. It is also a higher proportion than those found by Hindle and Snell for parish datasets, suggesting that pauper girls in London had access to a greater variety of formal work placements than those elsewhere.^31^ Krausman Ben-Amos and Simonton have both found that although young women were active participants in the labour market, they were much less likely to be formally apprenticed than boys. The terms of the Statute of Artificers were not gender specific, but women were more likely to receive training informally as children or widows, or to work in unskilled and casual employment which did not need an apprenticeship.^32^ This means that girls and young women remain significantly under-represented in discussions of work and training, making their high-profile presence in the current dataset all the more noteworthy. We cannot always be sure about what exactly they were being trained to do, as opposed to the profession of their masters. The training itself may have been given by the master's wife, and it may have included domestic service as well as, or instead of, the imparting of skills.^33^ Nonetheless, the dataset clearly shows that girls as well as boys were formally bound out, indicating that their legal status was also key for parish officials, and tied to the same overall framework as that for boys. This is especially noteworthy given the ubiquity of yearly contracts to domestic service in London. The high profile of girls in this dataset brings into question Snell's argument that they were squeezed out of the employment market around the turn of the century.^34^ The dataset also shows the formal participation of women in workplaces and training by including some mistresses named in their own right, or with their husbands.

## II

If parish authorities were indeed keen to get young people off the relief rolls and into employment, as has been implied in the literature, we would expect their apprentices to be younger than those bound by their families. Previous work has tended to show that this was indeed the case.^35^ There must have been a benefit to masters as well as parish officials, however, or they would not have accepted parish apprentices (although masters could be forced to take apprentices this seems to have been rare in practice).^36^ Wallis and Honeyman have both suggested that young children could be of some use in a factory or business, and that any lack of skill at the early stages was recompensed by the long period of the term.^37^ Humphries has also shown how the community surveillance of apprenticeship was joined to a system of deferred benefits for both sides in order to minimize early defaulting.^38^ Both masters' demands for labour and parish officials' desire to be rid of rate burdens encouraged the apprenticing of young children. Whether this could also reflect compassionate or altruistic aims is more debatable. Early apprenticing could have been a way to remove children from supposedly feckless parents, while giving them maximum opportunities to gain skills and experience. On the other hand, it also meant breaking up families, possibly against the wishes of the child's parents. Young ages at apprenticeship are not incompatible with a desire to build social capital and promote independence, but they sit less comfortably in a wider perspective on poor families as a whole.

As already noted, previous work has confirmed that parish apprentices were younger on average at binding than privately bound children. Snell finds an average age of 14.3 years for male apprentices who later made settlement examinations between 1700 and 1800, and of 13.5 years for females, while Lane also noted that Warwickshire non-parish apprentices tended to be approximately 14 years old. Both note that parish apprentices were younger (Snell cites one sample of parish boys with an average age of 13.4 years), and Sharpe has found children as young as 10 being bound by Colyton parish.^39^ Bindings at younger ages than this seem to have been rare, although it is clear that they did happen occasionally.^40^ The 1814–15 parliamentary *Report from the committee on parish apprentices* showed that less than 1 per cent of the 3,789 London parish apprentices bound to tradesmen, watermen, sea service, and household employment between 1801 and 1811 were under the age of eight, and that almost half were between the ages of 12 and 14. Only just over a quarter in total were under 12 (26.5 per cent).^41^ Among those sent outside London, however, more than two-thirds were under this age (68.8 per cent), raising the possibility that children bound beyond the metropolis were subject to different systems and considerations than those kept in the city. The precise age may also have varied according to the type of employment, with low-skilled and labour-intensive trades taking younger children, while the circumstances of individual children and families probably also directed the occasional placement of the very young.^42^ Children bound to certain sectors or locations were thus younger than the average for parish apprentices, as the popular picture suggests, but generally speaking, it is rare to find bindings of children under the age of eight or nine.

The current dataset supports this general consensus that parish apprentices were younger than those privately bound, but were not very young children. Both mean and median ages of those in this sample were 12 years (the mean value was 12.3), with the concurrence suggesting that it was not particularly affected by the age at which the child came under the parish's care. Girls were slightly older than boys, with an average of 12.5 years, compared to 12.0 for boys. This difference between the sexes was statistically significant at a 99 per cent level of confidence.^43^ Honeyman finds a variety of average ages among children bound to factories and mills, with many aged 10 or younger, although not much younger.^44^ This reinforces the findings from the contemporary data cited above, that factories did employ children at the lower end of the age spectrum. It will be interesting to see below whether this explains the younger average age at binding for boys. While factories do seem to have had a need or preference for young children (or perhaps more to the point, a long period of cheap service), there is very little data to suggest that we should be thinking in terms of large numbers of workers much under the age of 10 years. This differentiation by employing sector will be examined in greater detail below, permitting a finer reading of any differences across the wider group.

**Figure 2:**
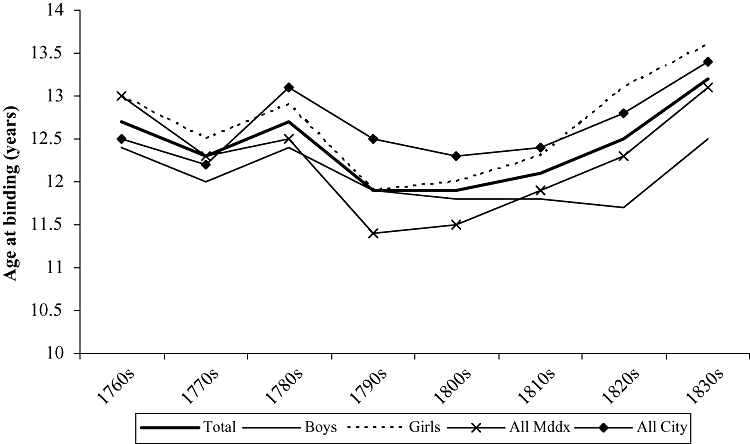
Average ages at apprenticeship, by decade and by location, 1760s to 1830s *Notes:* Total N is 3,166 (those missing from the 3,285 total in the sample lack age information); boys 1,815; girls 1,348; all Middlesex 1,755; all City 1,411. *Source:* Apprentice database.

An examination of average ages over time gives an early indication of a change in demand or employment type. The trends for the total sample, and for several sub-samples within it, are illustrated in figure 2, with the underlying data shown in table 2. The total sample shows some slight variation in the early decades, but there was a sustained lowering in average ages in the 1790s and 1800s, when the mean value dipped below 12 years. This was also a period of rising poor law expenditure, which brought an increasingly pressing demand to reduce the rate burden, and of full employment which increased the demand for hands.^45^ The lowering age over the turn of the century may thus be an indicator of changed demand for child labour, but it is likely also to reflect wider trends in poverty and poor relief. Average ages remained at this lower level until the 1810s, with the following two decades seeing a rise of a little over a year, although this was not statistically significant.^46^ The slight rise may be related to (and possibly anticipated) an act of 1816 which mandated that London parish children had to be over the age of nine at binding, although Honeyman finds evidence that the terms were not necessarily complied with.^47^ We should be also aware that some of the trends captured here could be partially due to the changing composition of the parishes represented in different decades.

**Table 2:** Average ages at apprenticeship among parish children, by decade and by location, 1760s to 1830s

	Total	Boys	Girls	All Middlesex	All City
1760s	12.7	12.4	13.0	13.0	12.5
1770s	12.3	12.0	12.5	12.3	12.2
1780s	12.7	12.4	12.9	12.5	13.1
1790s	11.9	11.9	11.9	11.4	12.5
1800s	11.9	11.8	12.0	11.5	12.3
1810s	12.1	11.8	12.3	11.9	12.4
1820s	12.5	11.7	13.1	12.3	12.8
1830s	13.2	12.5	13.6	13.1	13.4
Total	3,166	1,815	1,348	1,755	1,411

*Note:* Incomplete details on some entries mean that not all children in the sample can be assigned to a category. This explains the discrepancy between the total given here and the total of 3,285 in the whole sample.

*Source:* Apprentice database.

Figure 2 also shows that ages at binding may be further refined via a breakdown by originating parish. It reveals that apprentices from City parishes tended to be older than those from Middlesex, especially in the middle decades of the period when the difference was up to a year. These differences were statistically significant in all decades between the 1780s and 1820s.^48^ This is a revealing suggestion that parishes did not have the same practices when it came to apprenticing poor children, and that aims and practices may have differed at a micro level. This is a consideration that will repay further investigation below, but the received wisdom on age and employment type suggests a link with a greater use of bindings to factory industry from Middlesex parishes. In both geographical areas, girls were on average older than boys, and this achieved a high level of statistical significance in all decades except the 1760s (when numbers are small), and the 1790s and 1800s when the trend converges.^49^ The individual parishes do show some differences in average ages as well, and it is difficult to tell whether this derives from changes in apprenticing practices, or from local opportunities and aims. St Clement Danes and St Luke Chelsea had the lowest average age at binding, at below 11.5 years, while St Sepulchre Holborn (Middlesex division) had an average age of 13.1. The two parishes with the earliest start dates in their datasets fall in the middle of the group, which indicates that average ages are not straightforwardly related to period of coverage. The trends illustrated here indicate that average ages at binding were not constant over time, or by originating parish. The extent to which this may be related to the type of work apprentices were bound to do will be considered next.

## III

The examination of employment types moves to the crux of the investigation of parish apprenticeship. It will demonstrate *how far* this type of training fed into both traditional and developing sectors of the economy, and what this implies about the motivations of parish officials and employers alike. This, in turn, will permit a consideration of the role of London in provisioning different types of labour markets, a topic which will be further explored in the subsequent section on geographical patterns of apprenticing. To the former end, the trades to which the parish apprentices were bound have been classified according to the Booth/Armstrong scheme of occupations, in order to build up a schematic view of sectoral preferences and opportunities for child labour.^50^ The general problem of differentiating between dealing and manufacturing sectors inherent in this classification system is present to some degree here, but a more significant proviso is that we cannot always be sure that the child was going to be trained in the master's business.^51^ It has already been noted that this is a particular problem for female apprentices, who were likely to have been employed in housewifery as well as, or instead of, being trained in the master's named craft or trade. Some registers do, however, make the distinction clear by stating the girl's occupation as well as the master's. Erickson also suggests that girls bound to freemen of City companies were generally being trained in the master's profession, and it seems likely that this was at least partly true for the majority of apprentices in the current dataset as well.^52^

The most telling category in terms of interactions between parish apprenticeship and economic sector is manufacturing, since this is where the driving force behind economic growth was situated in this period. Manufacturing, however, took place in both the traditional and developing sectors of the economy, as will be noted. As table 3 illustrates, 76 per cent of bindings in total were to masters who worked in manufacturing industries (and a further 2 per cent in hybrid categories which included manufacture, such as ‘mantua making and domestic service’). This was a notably larger proportion than that found in the wider populations of many London parishes, a clear indicator that it was a disproportionately heavy employer of parish apprentices, and that their employment patterns did not simply map on to the profile of the apprenticing parish.^53^ This is indicative of an information network which extended beyond the parish itself, and the immediate trade and manufacturing contacts of local residents. Dealing and domestic service accounted for a further 4.5 per cent of bindings each, both sectors which Schwarz has noted were distinctively large in London in the mid-nineteenth century.^54^ Their small presence in the current dataset is another indication that parish apprentices did not participate in the particular employment profile of their home city. It is interesting to note that both domestic service and agriculture were named as occupations for small numbers of children, given that both also employed large numbers of young people on shorter, waged contracts rather than apprenticeships. This is further proof that parish officials remained committed to a traditional framework of training for the young, even when alternative contracts existed.^55^ There were only small numbers of children in the remaining occupational categories.

**Table 3:** Occupational classification of parish apprentices, 1751–1833

Occupational category	N	%
Agriculture	100	3.0
Building	77	2.3
Dealing	149	4.5
Domestic service	149	4.5
Industrial service	5	0.2
Manufacture	2,508	76.3
Mining	34	1.0
Mixed categories	74	2.3
No information	46	1.4
Personal and professional	69	2.1
Rentier*a*	23	0.7
Transport	50	1.5
Unknown	1	0.03
**Total**	3,285	

a *Note:* The term ‘rentier’ refers to the master in cases where no indication was given of the work the apprentice was to do (for example, ‘gentleman’)

*Source:* Apprenticeship database.

Further nuances can be added to this picture by breaking down the occupational profiles among parish apprentices by sex and parish of origin. This will again suggest whether different parishes participated in different employment sectors, and thus whether they used apprenticeship in different ways, either de facto or by design. The largest single area of employment, manufacture, showed very little difference between the sexes: 75.9 per cent of boys and 76.8 per cent of girls were bound to this economic sector. Children of both sexes were apprenticed to a wide variety of trades and industries with considerable overlap, but girls were found in a smaller range of manufacturing industries (186 different categories compared with 264 for boys) and were more heavily involved in textiles. Three-quarters of the girls went into textile industries compared with just under half of boys. The degree of overlap in manufacturing type supports Schwarz's assertion that there was not a great deal of sexual division in labour among parish apprentices at the turn of the century, although the pool was more limited for girls.^56^ The bias towards textile manufacture for girls is in line with the profile of adult women workers in London, and also reflects Lane's finding that textiles (including factory-based manufacture) predominated among production industries that relied on child labour.^57^ The remaining categories are small when broken down by sex, but we may note in passing that, as might be expected from adult working patterns, girls were more evident in dealing and domestic service sectors than boys, while the latter predominated among the small numbers going into agriculture, mainly bound to fishermen in Essex, Surrey, and Kent.^58^ The relationship between this finding and the average ages at binding will be explored in greater detail below.

There is more evidence of patterning in the occupational profile of parish apprentices based on parish of origin. Although the manufacturing sector predominated for City and Middlesex parishes alike, the latter made use of a greater diversity of employment types. City parishes, however, used manufacturing overall more intensively as an employer of pauper children. In this sector 81.9 per cent of City apprentices were bound to masters, compared with 74.0 per cent of Middlesex apprentices (not shown). The difference between these proportions was significant at a 99 per cent level of confidence.^59^ The Westminster parish of St Margaret and St John had the lowest proportion of all in manufacture, at 47.3 per cent, which drove down the average for Middlesex. Dealing occupations had a higher profile for the Westminster parish apprentices (7.4 per cent of the total) than either the City (4.2 per cent) or the rest of the Middlesex sample (1.4 per cent). Again, this was driven by St Margaret and St John, indicating that Westminster parishes were not all of a type.^60^ This preference for apprenticeships in dealing from a wealthy and fashionable parish may indicate a wider network of contacts based on local business links, or else a higher proportion of placements in the parish itself. The latter hypothesis will be tested in the following section. The City also contained concentrations of wealth, but rich families were tending to move out of the intramural area by the second half of the eighteenth century, perhaps lessening the need for service employments in the City compared with Middlesex and (especially) Westminster.^61^ While the City contained much small-scale industry, guild and company controls probably kept pauper children out of employment there. There is almost no evidence for parish apprentices being bound via City company indentures, a fact which must relate at least partially to their young age.^62^

**Figure 3:**
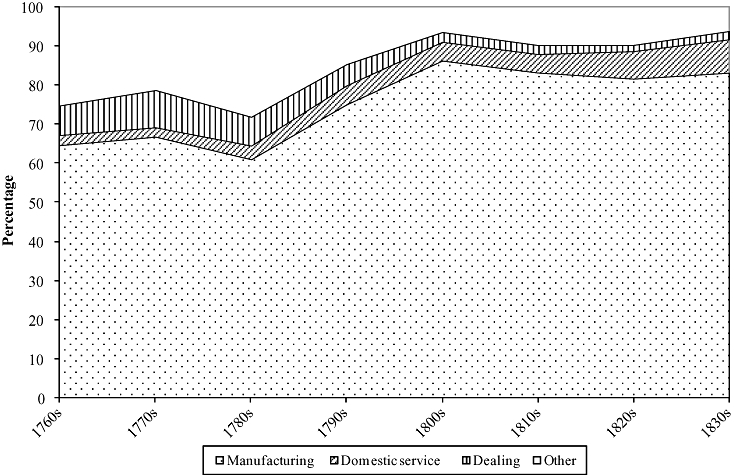
Breakdown of occupational classification of parish apprentices by decade, 1760s to 1830s *Source:* Apprentice database.

It is clear that a large majority of London's parish apprentices were bound into manufacture, although this varied by area. It remains to be seen, however, how far this related to the growth of the industrial sector in particular, and thus whether the function or economic impact of parish apprenticeship was changing over time. We may probe this further by examining whether there was any change in the distribution of apprentices across the occupational range as the period of early industrialization progressed. Figure 3 shows the proportion of apprenticeships in the largest occupational sectors (manufacturing, dealing, domestic service, and all other) by decade over the period. Some caution must be exercised in the interpretation of the data because of the way that individual parish datasets begin and end at different points. In terms of general trends, however, the exercise is still a useful one.

Figure 3 indicates that the proportion of parish children apprenticed into the manufacturing sector did increase from the 1780s, and that it subsequently remained high. No additional parishes were included in the dataset over the course of the 1780s and 1790s, making it likely that this was a real change rather than one due to the changing composition of the sample. The dealing and ‘other’ sectors shrank correspondingly, while domestic service increased its share. This latter trend is a noteworthy illustration of the continued importance of ‘traditional’ rather than ‘industrial’ occupations, although it might also reflect an increased tendency to specify the nature of girls' activities as much as a real change in occupational profile. Also potentially relevant is the legislative restriction passed in 1816 on the distance that London parish apprentices could be sent for work, which might have halted some bindings into distant manufactories and increased the relative importance of domestic service. Again, however, Honeyman highlights that government restrictions were not always observed.^63^

This pattern over time is also further nuanced when the sexes are considered separately. Given the uncertainty surrounding what parish girls were actually trained to do, it is particularly instructive to consider boys alone. The overall pattern by sector is similar to that found for both sexes.^64^ At the start of the period, however, boys were less likely than girls to be engaged in manufacturing trades, instead featuring in the ‘other’ sector. In the 1760s, 56 per cent of male bindings were in manufacture, compared with 76 per cent for girls. Boys were more likely to be bound into the agricultural and personal and professional sectors in these early decades of the period than later. The high point for boys' participation inmanufacture was in the 1800s, while it was a decade later for girls. This difference between the sexes in terms of participation in manufacturing was significantly different in both the 1800s and the 1810s.^65^ The relative prominence of the dealing sector early in the period under consideration was driven by girls rather than boys, as was the later increase in the proportional significance of domestic service. Boys remained represented across a mixed economy for longer than girls, although they subsequently became more concentrated into the manufacturing sector. Girls remained better represented in domestic service and dealing, although the latter had fallen away by the 1790s and did not recover.

Manufacturing thus loomed large in the experiences of work for pauper children, but what did this mean? Motivations and experiences may have differed quite substantially between manufacture in a factory, and in a small workshop of the type that London had in abundance.^66^ They also have very different implications for the role played by the capital in encouraging an industrial workforce in the regions, and in the underlying motivations for apprenticeship.^67^ Were parish apprentices being harnessed to a Smithian process of unskilled specialization driving further economic growth, or in the perpetuation of economically less efficient but skilled manufacture? Were those processes taking place in factories or small workshops, and what do they tell us about the value of apprenticeship for a child's ongoing employment and acquisition of skills? These are questions of crucial importance for establishing the role and importance of parish apprenticeship in the period of early industrialization.

We can begin to answer some of these questions by considering the types of work being undertaken in the prominent manufacturing sector. We have already seen that children bound to manufacture participated in a wide range of occupations. Across the period, however, the most common single type of manufacturing work for parish children was in cotton production (generally specified simply as cotton manufacture), with 467 children being apprenticed to this area (14.2 per cent of the total), generally in large groups, and a further 57 being bound to ‘worsted and cotton manufacture’. The single most prominent part of manufacturing in this context was clearly one attached to the burgeoning industrial sector. The next most popular category was the silk manufacturing industry (silk weaving, throwing, warping, and dying), which accounted for 247 bindings involving a relatively small number of masters. This indicates that each had a need for considerable numbers of child hands, pointing again to large-unit production, if not in a sector quite so forcefully associated with industrial growth. Honeyman shows that the largest textile employers of children of the firms she traced received 200 parish apprentices or more between *c*. 1785 and 1815. Others took much smaller numbers, but some of the larger employers, including John Birch in Backbarrow, Lancashire, made regular appearances in the current dataset.^68^ This prevalence of cotton and silk manufacture is similar to the pattern reported by the 1814–15 parliamentary inquiry into parish apprenticeship.^69^

So far, then, the evidence does point to a considerable presence of apprenticeship within large-unit manufacture. The next most popular categories were of a slightly different nature, however: 179 bindings to boot- and shoemaking and finishing trades, 174 to cordwainers, and 167 to weavers. Individual parishes also maintained links with specific small-unit industries. The most popular manufacturing trades in St Margaret and John, for example, were tambour-working/tambour-embroidery and cordwaining. While we should not assume that cotton and silk manufacture always took place in factories (although the scale of machinery made it increasingly likely), boot- and shoemaking and cordwaining were significantly less likely to do so. Clapham points out that single masters working with just one apprentice or mate, but owning their own tools and materials, were common at the lower levels of most trades in London.^70^ This is further evidence of the degree to which parish apprentices supported both traditional and industrializing sectors of the economy. The following section will examine how far this translated into participation in distinctively metropolitan/non-metropolitan economies.

At this stage we can push the investigation a little further by examining the relationship between individual parishes and the manufacturing sector, and whether these changed over time. The evidence shows that the Middlesex parishes were more likely to bind children into the cotton industry than those in the City. This again points to a different range of outcomes for parish children, which probably related both to pragmatic concerns to take up opportunities for apprenticeship wherever they lay, and connections with newly emerging industrial employers. St Clement Danes sent particularly large numbers of children into this sector (290), with St Luke Chelsea and St Leonard Shoreditch also participating. The City parishes, in contrast, rarely bound children to cotton manufacturers, instead using placements in the more traditional manufacturing sector. Manufacture in the boot and shoe trades, for example, was dominated by City apprentices: 137 out of 179 such contracts. Whether these preferences related to the range of occupations in the parishes themselves or the nature of links to employers further afield remains to be clarified. Parish officials rarely recorded their reasons for pursuing particular policies, and so a consideration of their own motives must remain speculative. However, it seems likely that a combination of opportunity, longstanding tradition, concern for pauper children and their future work prospects, and the inclinations and initiative of individual officers and employers all played a part. The questions asked of factory masters as to the future employability of parish apprentices demonstrate a concern for skill acquisition and security, but it is hard to believe that the opportunity to dispatch large numbers of children with relatively little effort was not also significant.^71^ This could take the form either of large-scale factory bindings, or of longstanding links with individual placements in traditional sectors. It is thus possible to read an ongoing tale of participation in both sectors of the economy into the patterns revealed here, and a diverse set of motivations from those in charge of parish apprenticeship.

Despite this apparent plurality of motivations, we do see some changes in the use of different sectors over the period under study. Across the board, bindings to the cotton industry were heavily concentrated in the 1790s and 1800s, with none at all taking place after 1819. This may be explained with reference to the 1816 ban on long-distance bindings from London, and changes in technologies, rather than reflecting a change in priorities.^72^ Bindings in the boot and shoe manufacturing trades, by contrast, show a surge in numbers between 1800 and 1829. The composition of the dataset may have had an impact on these patterns, although this risk is minimized by the fact that the data for St Clement Danes (the most prolific parish for bindings to cotton industries) extend beyond this period of intensive binding to cotton masters. In all cases, there is evidence for a retraction in the range of manufacturing sectors used for parish apprenticeship, from a large number of mainly textile-based crafts and industries in the 1770s and 1780s, to a smaller range in which cotton manufacture featured more prominently by the 1790s.^73^ After this, smaller scale manufacture was preferred again, possibly in response to a reduction in places available in distant mills and factories. If we read a reduced degree of concern for training and welfare into the use of bindings to the cotton industry, then this pattern reinforces Horrell and Humphries' statement that parish apprenticeship was moving towards ‘a harsher and less tolerant institution’, at least up to the turn of the century.^74^ While it would be unreasonable to think that all, or even some, parishes would turn down even exploitative opportunities for large-scale bindings when they were offered, this association still remains to be proven, however. Honeyman characterizes many parishes (including several in London) as protective of pauper children in industrial apprenticeships both in outlook and practice, making a change in practice not incompatible with continued concern for welfare.^75^

Furthermore, the current evidence illustrates the continued significance of manufacturing placements outside the industrial sector, even if the range contracted over time. The coincidence of this trend with the more competitive economic situation in London may reveal a story of small-unit business failure and reduced demand for manufactured goods. The continued use of manufacture in the traditional sector of the economy does not necessarily point to a greater commitment to human capital formation or child welfare on the part of parish officials either; indeed, it might indicate an unwillingness to seek out new and alternative methods of employment. Changes in legislative regulations further complicate how far the patterns revealed here are indicative of changes in demand in different sectors. The point is that parish apprenticeship did serve both traditional and industrial sectors of the economy even in the manufacturing sector, and that neither can be read as a straightforward commitment to the acquisition of appropriate work skills or demand for child labour in a changing economic climate.

## IV

As a final test of the nature of poor apprenticed labour, we now turn to a consideration of the locations to which the children were sent to work. This provides the final piece of the puzzle as to whether children's participation in employment changed as local industrial economies grew in certain parts of the country. This in turn illustrates the role of London in providing labour for those regional economies. In particular, we will be able to examine whether employment in cotton manufactories implied placements in the rapidly industrializing areas of the country, and whether a closer relationship with small-unit manufacture corresponded with placements nearer to London. The findings will be interpreted in the light of their implications for the value and purpose of parish apprenticeship.

Locating where children were placed is not straightforward, however. Officials frequently recorded only the master's street address with no identifying parish, and we cannot assume that this always meant that the master lived in the home parish or in a particular part of London. Different officials may also have recorded addresses with different degrees of detail. It is therefore particularly difficult to be accurate about the children who remained in London, although we can be reasonably secure in the identification of placements beyond the metropolis. Even with these caveats in mind, however, we see that a large minority (and probably the largest single category) of parish apprentices were placed with masters in the parts of Middlesex surrounding London. Of those who could be fairly safely counted as Middlesex placements, 50.6 per cent originated in the City, indicating that links extended beyond the nearby metropolitan parishes. Indeed, the most popular single receiving parish in Middlesex was St Matthew Bethnal Green, which was still relatively rural at this time, particularly on its eastern side, although notoriously impoverished and home to several industries, most notably, silk- and boot- and shoe-making.^76^ Several City parishes including St Botolph Aldgate farmed out their poor in this area, and in total, 60 per cent of apprentices placed in both Mile End and Bethnal Green in this dataset originated in the City.^77^ This indicates that parishes exploited existing links with the poor law machinery. It has already been noted that opportunities to apprentice City children within the parish were probably limited by guild restrictions, while a failure of local industry to provide sufficient employment opportunities and a desire to ensure settlement elsewhere probably also played a part. The importance of the local labour market should not be understated: Honeyman has highlighted that parishes generally did apprentice their children locally when opportunities were available, even when factory work is singled out.^78^

If parishes were keen to pass on responsibility for their poor children elsewhere in order to reduce the risk to the rates in the future, then we would expect to see reluctance on the part of the receiving parish to receive them for the same reason. Even if parish officials could not prevent the entry of pauper children, they might put pressure on householders to refuse to agree to indentures by threatening the removal of their own right to relief. This may have happened to some degree, but we do see children moving not only outside the home parish, but even into other parishes with many pauper children to bind out. Masters in St Luke Old Street, St James Westminster, and St Martin-in-the-Fields, for example, all took on parish apprentices from other parishes. Examples internal to the current dataset can also be found: one child was apprenticed from St Clement Danes to St Margaret and St Johns in 1818, and six were contracted in the opposite direction in the 1780s and 1790s. This suggests either that poor law officials were not able to prevent this inward traffic (although other requests may have been refused), or that parish apprentices were not seen as potential rate burdens, but rather as productive units of labour. The risk to the receiving parish would have been minimized by the need to complete an apprenticeship to guarantee a settlement, however, by which time the individual had acquired significant work skills. Certainly, this transfer or exchange of children from one London parish to another undermines the emphasis placed on the need to move potential rate burdens on.

This feature of parish apprenticeship would be further elucidated by an examination of how common it was to be apprenticed in the home parish. Unfortunately, it is almost impossible to establish the number of children who stayed, given the lack of precision in London addresses in the registers. St Leonard recorded at least 66 of its own children remaining within the parish, but it is again hard to know whether this reflected parish policy, opportunities for child labour, or the incidence of locally resident kin and friends. A count of children apprenticed to masters and mistresses of the same surname reveals a possible 37 contracts with family members, and a further 11 were noted to be bound to uncles, step-parents, and brothers. Of course, other family members could have different surnames, while some of these matches might be coincidental. In only two of these cases did the master live in the apprentice's home parish, however, suggesting that while the existence of local kin might be an explanatory factor for a small number of apprentices, it did not necessarily map on to the boundaries of particular parishes.

There is also evidence of relationships between London parishes and particular areas at a greater distance from the metropolis. The Westminster parish of St Margaret and St John, for example, had a preference for neighbouring counties, particularly Lambeth and Southwark in Surrey. The parliamentary investigation in 1815/16 into parish apprenticeship also noted that this parish (like others in Westminster) had not sent children at a distance from London since 1803.^79^ St Sepulchre Holborn (Middlesex division) also sent a large proportion of its children to the counties surrounding the metropolis (23.4 per cent). St Leonard Shoreditch, in contrast, sent only 2.5 per cent of its apprentices there, and St Clement Danes sent 10.4 per cent.^80^ The parishes that were under-represented in this region frequently used significantly further-flung areas instead. Children who travelled these greater distances were generally placed in northern cotton mills, confirming the association between distance and participation in the newer industrial sectors. St Clement Danes, for example, which sent a large proportion of apprentices into cotton manufacturing, also entered into a significant number of contracts with masters at a distance from London, with 46.3 per cent (292 children) of St Clement's apprentices being sent on lengthy journeys to their masters. St Luke Chelsea and St Leonard Shoreditch also used long-distance bindings frequently, while St Margaret and St John, St Giles Cripplegate, and St Botolph Aldersgate rarely did so. This is clear evidence that parishes provided labour for quite different parts of the economy, and that this might bring quite different assumptions on distances travelled as well as the nature of the work undertaken.

The most common of the outlying receiving places for parish apprentices was Lancashire. Of St Clement's 292 long-distance apprentices, 228 were bound to masters there, with 188 going to the cotton manufacturer John Birch in Cartmell. A further 55 St Clement's apprentices were sent to Glasgow, four to North Wales, three to Yorkshire, and one to Northumberland.^81^ Of all these children, only two were not placed with cotton manufacturers: one went to a mariner in Newcastle, and another to a boot binder in Lancashire. This particular parish clearly had a developed relationship with several industrialists who took large numbers of indentured apprentices to work in the textile industries. Indeed, the figures on business size cited above suggest that some parishes may have been supplying all of a factory's needs for child labour. The majority of these apprentices were boys: 63.4 per cent of those sent to Lancashire.

Apprentices from St Luke Chelsea show a variant on the same pattern. There, 130 children were apprenticed at some distance from London, of whom 39 were sent to Lancashire, all to the same master to be trained in cotton spinning, and 45 to Nottinghamshire, all to the same worsted and cotton manufacturer. Twenty-four children were bound to be miners in Bilston, Staffordshire, although several masters' names were given. Twenty of the Nottinghamshire apprentices were girls; all of those bound to Staffordshire and Lancashire were boys.^82^ It seems that at least some masters preferred to take parties of children of the same sex, perhaps to ease the practicalities of accommodation. The average age for these children was low, indicating that the reasons for seeking out child labour may have differed here. Children bound to manufacture far from London were, on average, 11.0 years old at apprenticeship, significantly below the 12.3 plus years for boys across the dataset (and even the 11.8 for boys in the 1800s and 1810s). This is closer to the average age found in Honeyman's sample of factory apprentices, and also confirms the association in other work between industrial occupations and young age which was discussed above.^83^ While younger children were probably little use as apprentices in skilled trades, they might have been of more value in a larger-scale workforce where the attainment of a certain level of all-round skill was less important. This would play into the classic view of deskilling in industrial manufacture, with pauper children assisting in its growth.

Of the 36 employers within the whole parish dataset who took five or more apprentices, all but one were from the manufacturing sector, the exception being a chimney sweep. In some cases the numbers involved were large; however, we should not lose sight of the fact that the majority of parish apprentices were bound in London or its vicinity, and that most were not part of the sort of mass bindings discussed here. Altogether, 22.1 per cent of all apprentices bound into the manufacturing sector were placed well beyond London, but more than half stayed in the City or Middlesex. Furthermore, mass bindings were not necessarily a facet only of the cotton mills; 36 children were placed with Thomas Flint, a trimming manufacturer in St Luke Old Street, and several women took in eight or nine female apprentices each for tambour working in Middlesex parishes. Large-scale bindings and industrial manufacture played a significant part in parish apprenticeship at this time, but it has been instructive to quantify its part in relation to smaller and more traditional economic sectors. Even within manufacturing, parish apprentices would have had a range of functions and experiences.

## V

The analysis reported here has quantified the breakdown of parish apprenticeship by economic sector during the period of early intensive industrialization. It has emphasized the significance of the traditional sector, and the ongoing use of placements local to London as well as the more high-profile mass bindings to the industrializing north. The current study has thus stressed the degree of continuity preserved in parish apprenticeship, which, although implicitly appreciated in the previous literature, has not been explicitly linked to the intentions of the old poor law and the traditional and local economy of the capital. Parish apprentices served both traditional and industrializing sectors simultaneously, with apparently little change in form or legal status. This picture has, however, been nuanced by the consideration of different parts of London: while some Middlesex parishes such as St Clement Danes clearly did cultivate relationships with factory industrialists at the turn of the century, others remained tied to smaller-scale manufacture close to London. City parishes made great use of placements in manufacture, but rarely bound children far from London, while some parishes in Westminster made greater use of sectors such as dealing, which reflected their own occupational profile. This points to a significant degree of variety in the experience and function of parish apprenticeship, and a need to consider the local situation on a parish level.

Although we cannot be sure of the intentions and views of parish officials on the role of apprenticeship, it has been suggested here that changes in its typology or function were not necessarily inconsistent with the continuity thesis.^84^ Honeyman's work has highlighted that parishes using factory bindings did appreciate the different work context they brought for children, for example, asking about conditions and employability. Whatever the economic context of the apprenticeship, however, officials were still concerned to provide for children and reduce their own outgoings, with the stress no doubt differing in different parishes. It seems unlikely that a parish that was consistently conscientious about its children would use placements that it felt represented a lesser investment in their young human capital as time went on (changes in personnel excepted). Conversely, a parish that was already negligent did not become more negligent in outlook by using factory placements. Once again, Honeyman's work has been invaluable for highlighting that parishes from all over the country made use of factory bindings: it was not an unusual occurrence and it did not necessarily tally with negligence or a lack of interest in providing for children.^85^ City parishes may have been able to use more local placements simply because they generally had smaller numbers of children to place, for example. The current study has moved this work on by contextualizing factory apprenticeship in the wider picture of pauper bindings, and stressing a continuity of aims across all sectors.

This view highlights the ongoing significance of training and settlement acquisition for pauper children, illustrated here particularly in its use for girls as well as boys. This is not to say, however, that the experience of apprenticeship did not change. The greater use of factory bindings in the industrial economy did mark several significant deviations from the longer history of parish apprenticeship. The first was the greater degree of mobility up to the end of the eighteenth century, and possibly beyond. Work on the earlier period has stressed the use of local placements, whereas the current study, like several others, has emphasized the long distances travelled by *some* children. This must have facilitated the development of the early factory labour force by providing large numbers of hands who would be subject to workplace discipline without the oversight of parents, and who could be formed into ‘early adopters’ of technology, to use the modern phrase. The second was the changed type of work that this might involve, particularly in respect of deskilling and monotony, conforming to a Smithian view of specialization which increases overall production. It seems unwise to over-emphasize the novelty of this change, however. It has been seen here that parish children apprenticed within the traditional economy tended to be bound to relatively impoverished textile and manufacturing trades which were not necessarily highly skilled. Even small workshops might divide processes into specialized stages, and it seems particularly likely that children would perform the simplest parts of any production process.^86^ They may not have been subject to the same regulation of hours and discipline as their peers in factories, but they did not necessarily escape the tedium and potential for being trained in only one part of a process which the Smithian model implies. Nor did they necessarily have any easier recourse to assistance if they experienced physical punishments. We should be wary of tying apprenticeship in different sectors of the economy into a binary view of training and deskilling. Similarly, we should not assume that one sector brought a greater investment in human capital than the other, especially when we bear in mind the ongoing emphasis on settlement acquisition and future work prospects. Children bound to the industrializing sector were arguably as likely to succeed in these respects as those apprenticed to traditional trades, while Humphries stresses that apprenticeship was vital in saving poor children from social exclusion.^87^ As long as their training prepared them for employment in that sector subsequently, human capital could be said to have been successfully formed.

The final theme brought out in this article is the degree to which the labour force of London parish apprentices shaped local economic development. It certainly supported a range of sectors in different parts of the country, and in certain cases provided substantial numbers of child workers for individual concerns. This, as Honeyman has stated, almost certainly helped to underpin the demands of a changed labour economy.^88^ What this study has also emphasized, however, is the degree to which individual parishes supported specific areas or industries to which they had links. Several of the City parishes, for example, provided apprentice labour for trades in Bethnal Green and Mile End where they also farmed out their poor, while St Margaret and St John sent children into Surrey parishes. This may have strengthened links with particular economic sectors and so reinforced investment in human capital in another sense. This is particularly true if it supported trades that supplied the London parish, or that strengthened its own demands for services.

Clearly, apprenticeship continued to play a significant role for pauper children throughout the old poor law period. The system of parish relief demanded that children, like other potential paupers, belong somewhere, and parish officials used apprenticeship as a means to safeguard settlements, move on potential rate burdens, and ensure a degree of training. This study has emphasized and quantified the variety of experiences this brought for children, but has also pointed out that this is consistent with a model that emphasizes continuity in outlook. Above all, it is now clear that factory labour was closely related to the aims, circumstances, and networks of individual parishes, and that the old poor law in London served the needs of both traditional and industrializing economies.

## Footnotes

2 For example, Rose, ‘Social policy’; Tuttle, ‘Revival’; Nardinelli, ‘Child labor’. These works have important points to make about the development of the factory workforce, and do not claim that it was the most important aspect of child work. Their focus tends to be less well related to the wider picture of parish apprenticeship as a whole at this time, however, and perpetuates the factory as the key site for juvenile labour.

3 For example, Rowley, *Poor relief*; Emmison, *Relief of the poor*, pp. 66–70. An early exception is George, *London life*, pp. 221–61. See also Kirby, *Child labour*.

4 Hindle, *On the parish?*, pp. 191–227; idem‘ “Waste” children?’.

5 Honeyman, *Child workers*; Humphries, ‘English apprenticeship’; Horrell, Humphries, and Voth, ‘Destined for deprivation’; Horrell and Humphries, ‘Child labour’; Horrell and Humphries, ‘Exploitation’.

6 Honeyman, *Child workers*, pp. 129–49; Humphries, ‘English apprenticeship’.

7 This runs counter to the portrait of parish apprenticeship found in the Webbs' account, which stresses bindings to factories as being of a different kind from individual contracts with masters (Webb and Webb, *Poor law history*, pp. 196–211).

8 For a good overview of the debates, see Mokyr, ‘Introduction’; also Hudson, *Industrial revolution*, pp. 9–36; King and Timmins, *Making sense*, p. 25; Berg and Hudson, ‘Rehabilitating’, pp. 30–1.

9 Mokyr, ‘Introduction’, p. 11.

10 Kirby, *Child labour*; idem, ‘Brief statistical sketch’. This feature of parish apprenticeship is noted also by Rowley in her study of Kinnerley in Shropshire (*Poor relief*, esp. pp. 6, 23), and Emmison's study on Eaton Socon in Bedfordshire (*Relief of the poor*, pp. 66–80).

11 Bolin-Hort, *Work*, p. 37, calling child apprenticeship ‘a cultural precedent for their recruitment to the early mills’; Rose (*Gregs*, *passim*, and esp. p. 10) also notes this continuity in labour management and sources of recruitment (in particular the workhouse) in early mills such as the Gregs' at Quarry Bank in Yorkshire.

12 See Wrigley, ‘Simple model’.

13 This is stressed by Humphries (‘English apprenticeship’, p. 74) as a neglected factor in apprenticeship during the early period of the industrial revolution. Honeyman, *Child workers*, pp. 138–9.

14 Hindle, *On the parish?*, pp. 9–11, 171–2, 192–218. He also notes that the institution was extremely controversial as it interfered with the family's own household economy, and implied that the poor could not train their children in diligence themselves.

15 Humphries, ‘English apprenticeship’; Honeyman, *Child workers*, pp. 113–28; Wallis, ‘Apprenticeship’.

16 Humphries, ‘English apprenticeship’, esp. p. 74; Rose, *Gregs*, pp. 26–8; Honeyman, *Child workers*, pp. 113–28.

17 On the development and structure of apprenticeship more generally, see, for example, Dunlop and Denman, *English apprenticeship*, pp. 28–60, 107–33; Snell, *Annals*, pp. 228–31.

18 Dunlop and Denman, *English apprenticeship*, pp. 248–60. On young vagrants and the fear of idleness, see Cunningham, *Children of the poor*, pp. 20–5.

19 Hindle, *On the parish?*, p. 210.

20 Humphries, ‘English apprenticeship’, pp. 97–8. This is also argued forcefully in Horrell et al., ‘Destined for deprivation’, with the old poor law characterized as alleviating the impact of inherited poverty and lack of human capital formation in deprived (particularly single-parent) families.

21 The so-called ‘heads of settlement’ were the conditions that gave a person legal entitlement to relief in a parish. See Burn, *Justice of the peace*, vol. IV, pp. 199–200, for an overview. See also Taylor, ‘Impact’. Rates of completion (and thus change of settlement) are very hard to access, especially when the individual did not take up the freedom of the city. Information on pauper children suggests a relatively high level of failure to complete, which would undermine attempts to pass on legal responsibility for them. See Krausman Ben-Amos, ‘Failure’; Wallis‘Apprenticeship’; Levene, ‘Honesty’. Snell (‘Apprenticeship system’, p. 308), however, notes that, after marriage, apprenticeship and yearly service remained the most common ways of gaining a settlement.

22 This made parish apprenticeship extremely controversial in its early days, with parents resentful of the implications that they were feckless and irresponsible, and the removal of the power of decision-making for their own families. Hindle, *On the parish?*, pp. 119–226, *passim*.

23 Schwarz's (‘London apprentices’) assessment of the demographic impact of London apprenticeship is based on Finlay and Elliott's estimation that they made up 4 to 4.8% of the population of the capital by 1700 (a significant lowering from the previous period).

24 For example, see Dunlop and Denman, *English apprenticeship*, pp. 28–60, 107–33; Snell, *Annals*, pp. 228–31; Brooks, ‘Apprenticeship’; Lane, *Apprenticeship in England*, p. 9. The most common reason given for the decline in apprenticeship more widely is that it was seen as increasingly unnecessary, with many trades taking place outside the area and remit of the City guilds. The rise of apprenticeship premiums also priced many parents out of the market, while young people were apparently increasingly reluctant to remain in unwaged work into their early twenties. Wallis (‘Apprenticeship’) stresses its ongoing importance throughout the preindustrial era.

25 42 Geo. III c.46 (1802). This was separate from the 1802 Health and Morals of Apprentices Act (42 Geo. III c.73) which has received greater attention.

26 George, *London life*, p. 59.

27 The omissions were those that were not identified in the first survey of archive holdings, or whose records were not in the regular format. St Pancras and St Martin-in-the-Fields are notable examples. Honeyman (*Child workers*, pp. 63–5) notes that the former made heavy use of factory placements and its omission from the current dataset may thus play down this aspect of pauper apprenticeship. No omissions were made on the basis of any defining characteristics that call the interpretation of the sample into doubt, however.

28 George, *London life*, p. 73. George also notes that the poor in Westminster lacked industrial employment, as the economy was largely based on service industries and trading. See also Harvey, Green, and Corfield, ‘Continuity, change and specialization’, pp. 469–73.

29 Schwarz, ‘Occupations and incomes’.

30 Landers, *Death and the metropolis*, pp. 312–15, including St Giles Cripplegate and St Sepulchre Middlesex. George (*London life*, pp. 78, 91) also notes the poverty and poor housing in several of these parishes.

31 Hindle‘ “Waste” children?’, p. 34; Snell*Annals*, p. 278.

32 See Brooks, ‘Apprenticeship’, p. 53; Schwarz, *London*, p. 22; Snell, *Annals*, p. 270 (see pp. 270–319 for female apprenticeship more generally); Krausman Ben-Amos, ‘Women apprentices’; Simonton, ‘Apprenticeship’, pp. 243–55.

33 It was hoped indentures would be more informative, but those sampled did not give further details on what the children were actually being trained to do.

34 Snell, *Annals*, p. 312.

35 Hindle, *On the parish?*, p. 213.

36 Ibid., pp. 192–3 and 198–203. This aspect of parish apprenticeship was the subject of much wrangling, but the *principle* of compulsion was gradually confirmed both in law and in practice. Webb and Webb (*Poor law history*, pp. 206–10) portray compulsion as being much more common after the Factory Acts started to limit how far children could be sent to work, and how long they could work for.

37 Honeyman, *Child workers*, pp. 113–28; Wallis, ‘Apprenticeship’. Wallis, however, notes that the ongoing balance of benefits for both sides breaks down for paupers and large organizations, where there is greater specialization and distance between apprentice and master. This implies that traditional apprenticeship and a phase of deskilling in the industrializing labour force are incompatible (reinforced by Adam Smith's opposition to apprenticeship regulation, which he felt restricted labour). The current analysis, however, reads a greater degree of continuity of experience between private and traditional parish apprenticeship in this respect, especially when masters took only one apprentice into a small workshop. In this case, their labour might have had ongoing value to the master, and was less likely to bring division of labour. Epstein (‘Craft guilds’) also extends the positive role of guilds and traditional craft apprenticeship into the period of early industrialization.

38 Humphries, ‘English apprenticeship’.

39 Snell, *Annals*, pp. 323–32; Lane, *Apprenticeship in England*, p. 13; Sharpe‘Poor children’, pp. 255–6.

40 For example, Honeyman (*Child workers*, pp. 45–6) finds that ‘children below the age of seven constituted a handful’ of those bound to Toplis & Co. worsted manufacturers in Nottinghamshire, although 11 were aged only five or six years.

41 *Report from the committee* (P.P. 1814–15, V). Other parliamentary reports on child labour also highlighted that children working in factories tended not to be under the age of 10, although parents often wanted to set them to work at a younger age. For example, see *Report of the minutes of evidence* (P.P. 1816, III). See also Nardinelli, ‘Child labor’, p. 742. Children put to work by their parents may not have been subject to the same lines of supply and demand as parish apprentices, however.

42 Unfortunately, the data do not provide this level of detail. See also Lane, *Apprenticeship in England*, p. 14.

43 Using a two-sample t-test.

44 Honeyman, *Child workers*, pp. 45–7.

45 Lees, *Solidarities*, pp. 83–4.

46 A fitted line of constant age passed through all the error bars marking one standard deviation from the mean for each decade. The null hypothesis of no change over time cannot therefore be rejected.

47 Honeyman, *Child workers*, pp. 51–3. Both Bolin-Hort and Horrell and Humphries specifically tie younger average ages to particular stages of industrialization. Bolin-Hort (*Work*, pp. 40–2 and 47–53) highlights the early stages of parish apprenticeship in factories; later on, methods of production shifted to place greater value on high-intensive adult labour in conjunction with greater mechanization and the employment of families. Horrell and Humphries (‘Exploitation’, pp. 496–8), using quite a different focus, pick out the 1820s and 1830s as the period when the ‘exploitation of *little* children’ (emphasis added) was most intensive.

48 At a 99% level of confidence (tested using the two-sample t-test).

49 Using a two-sample t-test. The results in the decades listed were significant at a 99% level of confidence.

50 Armstrong, ‘Use of information about occupation’. The categories are those in his industrial classification scheme (pp. 191–310), modified in accordance with Harvey, Green, and Corfield, *Westminster historical database*.

51 On the former problem, see Harvey et al., *Westminster historical database*, p. 72.

52 Erickson, ‘Identifying women's occupations’.

53 For example, Black (‘Putative fathers’, pp. 53–9) has found that while manufacture was the largest single occupational category among the putative fathers of illegitimate children in both St Leonards Shoreditch and St Clement Danes (mainly clothmaking, shoemaking, and tailoring), they constituted only 31 to 45% of all men named in bastardy examinations in those parishes. Furthermore, the size of the manufacturing sector may have been declining towards the end of the eighteenth century.

54 Schwarz, *London*, p. 13.

55 Yearly contracts may have been more often used for older children who would not need training and who could be more immediately productive. Apprenticeship rather than yearly service also gave children an occupation for a number of years, which may have been more appealing for overstretched parish officers. As is noted below, most of the placements in the agricultural sector were with fishermen rather than farm service.

56 Schwarz, *London*, pp. 20–1. This revises Snell's (*Annals*, pp. 278, 287–90) argument that the labour of parish apprentices was highly gender specific, although Schwarz's evidence shows that this may have been truer of earlier decades. Snell seems to ignore apprenticeships in service and collective industries, however, which were the most popular categories here.

57 Schwarz, *London*, p. 22; Lane, *Apprenticeship in England*, p. 169.

58 Dealing accounted for 7.2% of female apprenticeships compared to 2.6% of male ones (100 girls and 49 boys), with victuallers and greengrocers among the most common occupations. Other girls may have been employed in shops (an occupation which Campbell thought was not worthy of a lengthy apprenticeship; see, for example, Campbell, *London tradesman*, p. 188, on apprenticeships to grocers). Domestic service accounted for 5.4% of parish girls (75) compared with 3.9% of boys (74, eight of whom were trained by women). As already noted, an indeterminately greater number of parish girls may also have worked at least partly in domestic service, but were recorded only in relation to their master's occupation. Service was a very heavy employer of women in London; see Earle, *City full of people*, p. 82. Kirby (‘Brief statistical sketch’, p. 232) found that almost 59% of girls aged 10–14 who were recorded with an occupation in the 1851 census were employed in domestic service. Agriculture employed 4.9% of boys in this sample (94 cases) compared to 0.4% of girls (6 cases).

59 Tested using a z-test for two proportions.

60 Harvey et al., ‘Continuity, change and specialization’, p. 473. They find that the largest economic sector among male voters in Westminster elections involved making and selling consumer goods (pp. 478–9). This sector constituted 31.5% of male voters in 1784, which was significantly higher than the proportion of parish apprentices bound into dealing (478). Rogers (*Whigs and cities*, p. 181) also notes the distinctive features of Westminster's consumer economy.

61 George, *London life*, pp. 73–5.

62 Guild and company apprentices had to be 14 years old or older, which precluded most of the parish apprentices considered here (ibid., p. 223).

63 Honeyman, *Child workers*, pp. 52–3.

64 The null hypothesis that the trend observed between the two sexes was the same could not be rejected using a two-sample z-test.

65 Tested using the two-sample z-test. The remaining employment sectors contained too few children when broken down by sex and decade to allow for statistical testing.

66 On small-unit manufacture and the English economy, see Clapham, *Economic history*, pp. 72–4. See also Berg, ‘Factories’, p. 139, for a more varied picture.

67 Schwarz, *London*, pp. 4, 31–2; George, *London life*, p. 164; Harvey et al., ‘Continuity, change and specialization’. These authors suggest that further specialization was a means of promoting economic development. Beier (‘Engine of manufacture’) also argues for a greater prominence of small-scale production in early modern London than had often been thought.

68 Honeyman, *Child workers*, pp. 92–4. John Birch was the single largest employer of parish children in the current dataset (taking 188 apprentices in total).

69 The *Report from the committee* (P.P. 1814–15, V) shows that three-quarters of apprentices sent beyond London went to masters connected with cotton manufacture. Large numbers also went into silk, flax, and worsted manufacture.

70 Clapham, *Economic history*, pp. 166–72.

71 Honeyman, *Child workers*, pp. 132–40.

72 Rose, ‘Social policy’, p. 21. This contrasts with Honeyman's (*Child workers*, p. 89) finding that long-distance bindings continued ‘well after the first decade of the nineteenth century’. The difference may relate to the fact that her study scrutinizes individual businesses to a greater degree than is possible here, and also covers a range of parishes outside London. Bolin-Hort and Rose both note a diminishing role for apprenticed child labour in factories by the second decade of the nineteenth century, with the Gregs' Quarry Bank Mill being one of the very few to continue to use them in relatively large numbers (Bolin-Hort, *Work*, pp. 35–7; Rose, *Gregs*, pp. 54–7).

73 The most common manufacturing trades for parish apprentices in the 1770s were weaving, tambour working, cordwaining, perukemaking, and watch- and clock-making and finishing. Lane (*Apprenticeship in England*, p. 38) cites the clothing trades in particular as ‘dead-end’, overstocked ones with few career prospects, suitable only for orphans and the poor.

74 Horrell and Humphries, ‘Child labour’, p. 98.

75 Honeyman, *Child workers*, pp. 215–38. See also pp. 239–60 for a discussion of neglect.

76 Schwarz, ‘Occupations and incomes’, esp. pp. 93–4; Kirby, ‘Brief statistical sketch’, p. 230; see George, *London life*, pp. 187, 190, 192, on the impoverished nature of the area, its overstocking with apprentices, and its opportunities for female and child labour.

77 Guildhall Library, St Botolph Aldgate, Lists of poor sent ‘to farm’, 1800–19, MS 2,677. Murphy, ‘Metropolitan pauper farms’, shows that Bethnal Green and Mile End were prominent among locations for pauper farms of City poor.

78 Honeyman, *Child workers*, pp. 58–90, *passim*. Lane (‘Apprenticeship in Warwickshire’) finds this tendency to local recruitment in her study of two Warwickshire mills.

79 *Report from the committee* (P.P. 1814–15, V).

80 The average for this area was 15.2% of all apprenticeships.

81 Tuttle (‘Revival’, p. 59) notes that by 1833–4, 34% of the workforce of Lancashire cotton mills were children, with a similar proportion in Glasgow.

82 This may relate to the ease of housing only one sex, especially after the 1802 Health and Morals of Apprentices Act mandated that there be separate accommodation for boys and girls.

83 A contrast is provided in Rowley's work on Kinnerley, Shropshire, where a local factory master proposed taking parish apprentices from the age of 14, and directly employing those aged between seven and 14 (*Poor relief*, p. 23). This nonetheless supports the assertion that young children were of value to factory masters.

84 Rose (‘Social policy’) cites some discussion of attitudes towards child labour and underlying reasons for decisions in parish vestry minutes, while Hammond and Hammond (*Town labourer*, p. 54) note that other parishes fought the attempt to limit the distance apprentices might be sent. On the whole, however, it is rare to see discussion of views on child labour rather than simply the outcomes of decisions.

85 Honeyman, *Child workers*, *passim*.

86 George (*London life*, pp. 202, 223, 230, 234) emphasizes this point for many of the trades to which pauper children were bound, and also that they were subject to seasonal unemployment.

87 Honeyman and Humphries both state that apprenticeships in factories did give training in skills relevant to employment in the industrial sector; Honeyman, *Child workers*, pp. 129–49; Humphries, ‘English apprenticeship’, p. 74.

88 Honeyman, *Child workers*, pp. 260, 264.

## Official publications

*Report from the committee on parish apprentices* (P.P. 1814–15, V).

*Report of the minutes of evidence taken before the Select Committee on the state of the children employed in the manufactories of the United Kingdom* (P.P. 1816, III).
